# Derm dotting technique to detect micro-invasive component in lentigo maligna and subsequent margin control with a modified staged excision technique^؆^

**DOI:** 10.1016/j.abd.2025.501191

**Published:** 2025-08-09

**Authors:** Nelson Lobos-Guede, Pablo Vargas-Mora, Gabriela Coulon, Chantal Caussade, Valentina Darlic, Magdalena Delgado

**Affiliations:** a Head and Neck Surgery Department, Dermato-oncology Unit, Instituto Nacional del Cancer, Santiago, Chile; b Dermatology Department, Facultad de Medicina, Universidad de Chile, Santiago, Chile; c Dermatology Department, Clínica Alemana, Universidad del Desarrollo, Santiago, Chile; d Faculty of Medicine, Universidad de los Andes, Santiago Chile; e Faculty of Medicine, Universidad del Desarrollo, Santiago Chile; f Pathology Department, Clínica Alemana, Universidad del Desarrollo, Santiago, Chile

Dear Editor,

Detecting microinvasion in lentigo maligna (LM) is crucial for accurate staging and management. Various techniques have been proposed to mark areas of interest for histological analysis, but their use in LM is limited. Reflectance confocal microscopy (RCM) may aid in this process, though identifying microinvasive atypical melanocytes remains challenging. We describe a practical approach using the derm-dotting (DD) method to assess microinvasion while simultaneously controlling peripheral margins during surgery.

A 72-year-old male presented with a 2.5 cm polychromic macule on the nasal dorsum, showing progressive growth and color changes over the past year. An incisional biopsy confirmed LM, and he was referred for a complete peripheral margin assessment. Dermoscopy revealed two well-defined hyperpigmented eccentric areas with angulated lines, rhomboid structures, asymmetric follicular pigmentation, and dark blotches, interrupted by a central light brown zone with a pseudonetwork pattern. After discussion in our melanoma committee, we opted for a modified staged excision technique (MSET) as a “slow Mohs” procedure to assess 100% of the peripheral margins, instead of using paraffin-embedded radial sections. Before the debulking of the central portion of the melanoma, we decided to perform an in vivo DD technique. We selectively marked (with red nail polish) the dermoscopic areas of irregular dark blotches and pigmented follicular openings to ensure they were processed and examined under a microscope (Fig. 1B). We debulked the tumor and sent it for paraffin-embedded sections for prognostic assessment. For margin evaluation, we excised four peripheral 5 mm skin quadrants beyond the dermoscopic perimeter, marking each with a suture at the distal border, oriented clockwise (Fig. 1C). Additional sutures were placed for reference in case further excision was needed. Histology revealed invasive LM with a 0.42 mm Breslow thickness, no ulceration, and two positive margins (1 and 2) (Fig. 1D). The involved margins were immediately enlarged, all proving tumor-free. The defect was repaired with a full-thickness graft.

**Fig. 1:**
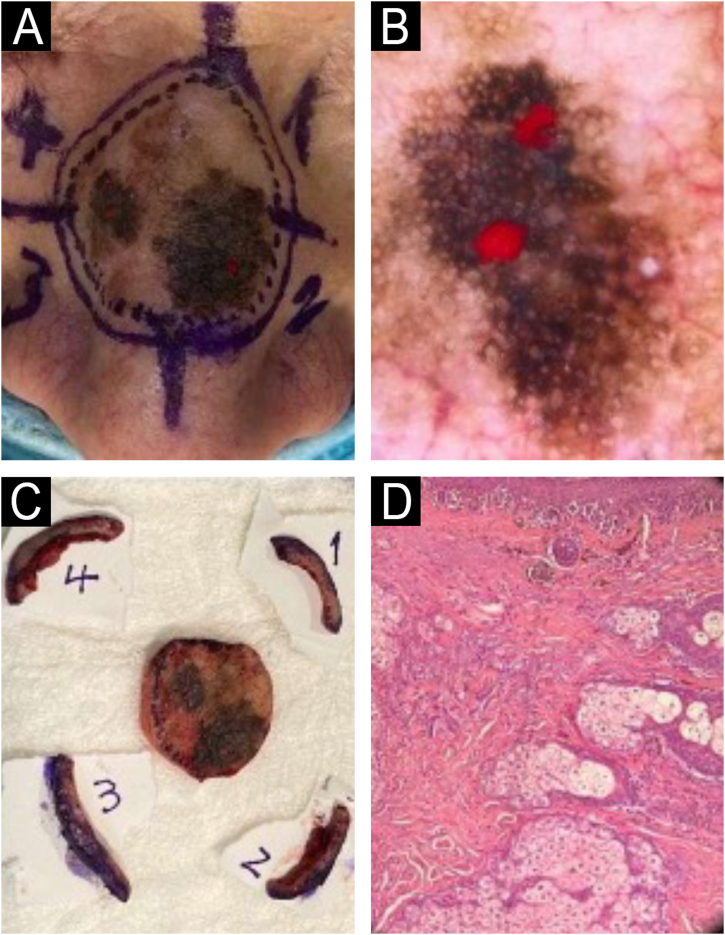
(A) Pre-operative clinical image of the dorsum nasi LM. (B) In vivo derm dotting image showing suspicious areas for invasive components on dermoscopy marked with red nail polish. (C) Tumor debulking of the central LM and four perimeter quadrants of 5 mm guided by dermoscopy. (D) Histopathology: Extensive involvement of melanoma in situ and a microinvasive focus of 0.42 mm Breslow (Hematoxylin & eosin, 10×, paraffin-embedded permanent sections).

Conventional skin biopsy processing may miss key areas between serial sections. Dermoscopic marking techniques enhance histopathological analysis by highlighting these regions.^1^, ^2^ For this purpose, punches, sutures, or nail polish have been suggested. The latter has the advantage that it is not altered during the processing of the specimen nor does it affect the histological evaluation. On the other hand, non-invasive techniques such as RCM or optical coherence tomography can be used for this purpose, however, their high cost and low availability make their use difficult.

Haspeslagh et al. using ex vivo DD in 8,584 biopsies, showed that the diagnosis of affected margins in non-melanoma skin cancer significantly increased with the use of this technique. So did the diagnosis of the degree of dysplasia in melanocytic nevi and the presence of ulceration in melanoma.^3^

The DD technique was originally described with in vivo dermoscopic marking, but there are also reports using the ex vivo modality.^1^, ^4^ For marking purposes in vivo DD has the advantage that certain structures, especially those of vascular origin, are not altered.

To our knowledge, to date, there have been no reports of the specific use of DD in LM to detect microinvasive foci. Peruilh-Bagolini et al. described irregular blotches and angulated lines as significant dermoscopic predictors of invasive LM.^5^ Like that reported case, we usually use these findings to indicate the use of DD and find a positive correlation with the microinvasive foci.

DD can also allow us to make other diagnostic and therapeutic decisions since detecting microinvasive foci would allow us to rule out non-surgical treatments such as imiquimod. In addition, detecting invasive foci allows us to make decisions such as performing a sentinel lymph node.

In this case, we did not expand the peripheral or deep margins after identifying microinvasive foci, as these atypical cells were confined to the debulking center, with only in situ involvement at the periphery. The benefits of margin enlargement in microinvasive LM remain unproven, requiring further longitudinal studies. However, given the thorough histological assessment provided by MSET, its use appears reasonable, particularly for extensive facial lesions.

We present a simple, cost-effective dermoscopy-guided technique to mark areas of interest for histological analysis, aiding in the detection of invasive components. Simultaneously, MSET allowed for complete peripheral margin assessment, optimizing surgical time.

## Research data availability

Does not apply.

## Scientific Editor-in-Chief

Sílvio Alencar Marques.

## Financial support

None declared.

## Authors’ contributions

Nelson Lobos: Approval of the final version of the manuscript; critical literature review; manuscript critical review; preparation and writing of the manuscript.

Pablo Vargas: Critical literature review; manuscript critical review; preparation and writing of the manuscript.

Gabriela Coulon: Critical literature review; manuscript critical review; preparation and writing of the manuscript.

Chantal Caussade: Critical literature review; manuscript critical review; preparation and writing of the manuscript.

Valentina Darlic: Critical literature review; manuscript critical review; preparation and writing of the manuscript.

Magdalena Delgado: Critical literature review; manuscript critical review; preparation and writing of the manuscript.

## Conflicts of interest

None declared.
